# Peritoneal dialysis in Tunisia: complications, technique and patient’s survival (twenty-seven years of experience in a single center)

**DOI:** 10.11604/pamj.2021.39.179.29354

**Published:** 2021-07-07

**Authors:** Meriem Ben Salem, Amel Ayed, Sahbi Khaled Taieb, Insaf Handous, Manel Ben Saleh, Mouna Hamouda, Ahmed Letaief, Sabra Aloui, Habib Skhiri

**Affiliations:** 1 Department of Nephrology, Dialysis and Kidney Transplantation, Fattouma Bourguiba University Hospital of Monastir, Avenue Farhat Hached, Monastir, Tunisia

**Keywords:** Peritoneal dialysis, peritonitis, patient survival, technique survival

## Abstract

**Introduction:**

peritoneal dialysis (PD) is a renal replacement therapy method that offers various advantages to end-stage renal disease patients. The aim of our study was to analyze patient characteristics, peritonitis and clinical outcome over a 27-year period of PD in our center.

**Methods:**

retrospective study of incident patients on PD from January 1990 to December 2017. A total of 304 patients were enrolled in the study group. All patients over 15 years of age entering the dialysis program were included in the study. Patients dropping out from PD within three months were all excluded. Biochemical and demographic variables, peritonitis episodes and patient and technique survival were analyzed.

**Results:**

the PD prevalence in our center was 4.5% during the study period; the mean age was 46.47 ± 18.6 years; diabetic nephropathy was the main cause of chronic kidney disease: 35.5% (n=108). Cardiovascular disease was the main cause of death: 39.6% (n=34). The peritonitis rate was 0.68 episode per patient-year. Ultrafiltration failure was the most important cause of PD withdrawal: 43% (n=60). Occurrence of peritonitis was the only independent predictor of technique failure: adjusted relative risk [aRR] 5.07, 95% CI 2.69-9.58; p<0.001. The overall non-adjusted patient survival was around 100%, 95% and less than 20% at 1, 4 and 25 years respectively basing on the Kaplan-Meier analysis. The group undergoing renal transplantation had the best survival rate.

**Conclusion:**

peritonitis remains the most common complication as well as the most provider of technique failure and patient´s transfer to hemodialysis. The technique survival was better in case of absence of peritonitis. However, our series showed the superiority of hemodialysis over PD in terms of overall patient survival.

## Introduction

As the global burden of chronic kidney disease continues to increase, so does the need for a cost-effective renal replacement therapy. Peritoneal dialysis (PD) is a method that offers various advantages to end-stage renal disease patients. This therapy is expanding around the world despite the reluctance of some patients and doctors; it´s estimated in 2018 that the frequency of PD use approaches 11% of dialysis population worldwide [1,2]. In Tunisia, the first acute PD was performed in 1963. But, the introduction of the technique in Monastir occurred only in early 1990´s. Ever since, the number of patients beginning renal replacement therapy has increased [3]. The evolution can be enamelled with complications; the most serious and life-threatening complication remain the infectious peritonitis while impacting the technique survival as well as the mortality in PD. In this context, we had conducted our study aiming to analyze the epidemiology, patient characteristics and clinical outcome over a 27-year period of PD in a single center (Monastir). We were interested also to evaluate the complications occurring essentially peritonitis (prevalence, microbiological profile, potential risk factors involved and its impact throughout the period of follow-up).

## Methods

**Study design and settings:** this was a retrospective cohort study carried out in the Nephrology Department of Fattouma Bourguiba University Hospital (Monastir, Tunisia) enrolling all patients followed up in our PD unit between January 1990 and December 2017. Incidence of complications during the follow-up, while emphasizing upon peritonitis and exit site/tunnel infections, was determined. Technical survival was measured as the period in which the patient remain using PD technique. Dropout data till the end of the follow up were stratified as definitive transfer to hemodialysis (HD) for any reason (mechanical problem, ultrafiltration failure, infection etc), death, renal transplantation and recovery of renal function. Predictors of mortality as well as patient and technique survival rates were analyzed.

### Study population

**Inclusion criteria:** we were interested to patients diagnosed with end stage renal disease and choosing PD technique weather for personal, social or medical reasons. Those initiating PD in emergency or in a planned way were all included.

**Figure 1:**
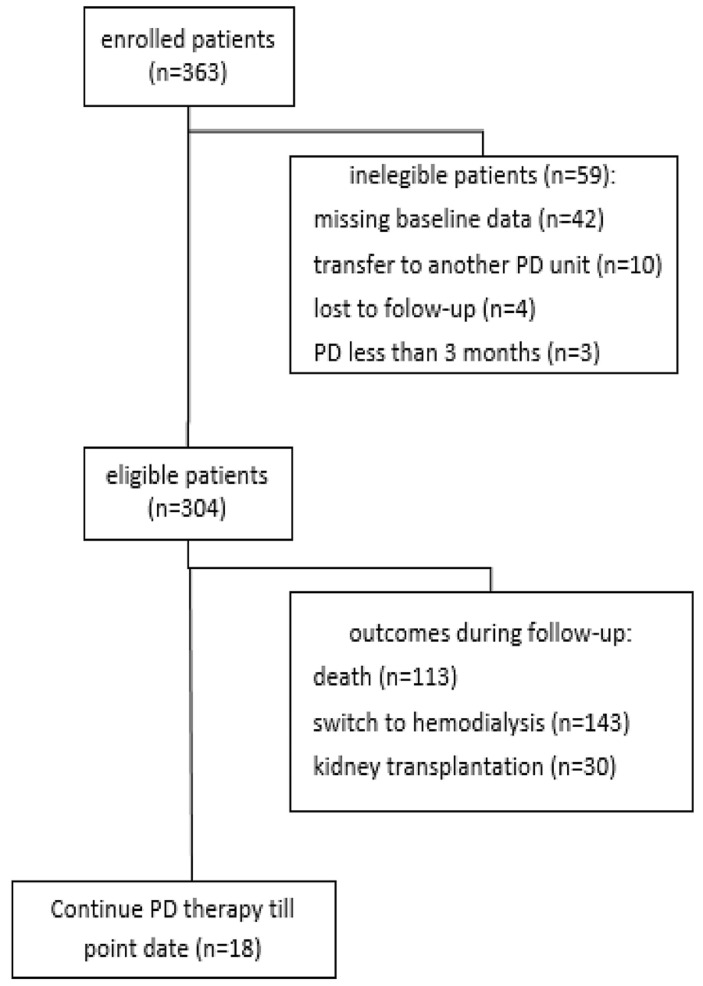
enrollment and outcomes of the population studied

**Non-inclusion criteria:** patients dropping out from peritoneal dialysis within three months, or having chronic renal transplant failure before initiating peritoneal dialysis, or benefitting from catheter placement in our service then being followed in another dialysis unit, or having incomplete baseline data, were all excluded. A total of 304 patients were enrolled in the study group. We divided our patients into four groups for better study of survival: group 1: patients using only peritoneal dialysis (132 patients); group 2: patients transferred to hemodialysis and remaining on it (127 patients); group 3: peritoneal dialysis patients then undergoing renal transplantation (30 patients); and group 4: patients transferred to hemodialysis then undergoing renal transplantation (15 patients) (Figure 1).

**Data collection:** an operating sheet, based on patient records and data from the French peritoneal dialysis registry, was established for each patient. Socio-demographic and clinical and laboratory data, including age, gender, educational and economic level, medical history, etiology of end-stage renal disease patients, nature of peritoneal dialysis decision and assistance modality were collected at the time of peritoneal dialysis initiation. We used the Charlson Comorbidity Index to evaluate the burden of comorbid systemic diseases.

**Statistical analysis:** statistical analysis were performed using the Statistical Package for the Social Sciences software (version 21.0; SPSS). The continuous quantitative variables were expressed as mean ± standard deviation (SD) or median and interquartile range (IQR) for skewed distributions. The categorical data were expressed as absolute values and proportions. The χ^2^ test and the Fisher´s exact test were used to assess non-parametric variables according to the application conditions and Student t-test was used to analyze clinical and biochemical parameters. Multivariate analysis using regression (binary logistic) was performed using covariates identified in univariate analysis and presenting a *p*-value lower than 0.2. Kaplan-Meier method was used to estimate the rates and plot survival curves of patient and technique survival; the analysis of the difference was assessed using the log-rank test. The difference between the variables was considered statistically significant if the *p*-value was less than 0.05.

**Ethical considerations:** no conflict of interest was declared. Ethical committee of Fattouma Bourguiba University Hospital was involved. The confidence of data was respected during the different steps of the study.

## Results

**Characteristics of study population:** the peritoneal dialysis prevalence in our center was 4.5% during the study period. A total of 304 eligible patients (predominantly males making up for 70% (n=211)) were enrolled. The mean age was 46.47 ± 18.6 years. Thirty six percent of patients (n=110) were diabetes and 15% (n=46) had a history of cardiovascular disease. The choice of the technique was guided mostly by patient preference (86%; n=262) while 14% of patients (n=42) made a compulsory choice to initiate peritoneal dialysis (vascular access exhaustion (n=15) or heart failure (n=26) or intolerance to hemodialysis (n=1)). The main causes of renal failure were diabetic nephropathy (35.5%; n=108) and chronic interstitial nephropathy (23.4%; n=71). The PD modality often used was continuous ambulatory peritoneal dialysis (67%; n=204). Over 37% (n=112) of the patients were assisted when performing PD. The baseline characteristics of the entire cohort are shown in Table 1. Our experience with the PD had known three different periods: 1990-2000, 2001-2006 and 2007-2017; the difference had concerned the type of catheter, the placement technique and time to start the exchange. Table 2 presents the full comparison of clinical and demographic characteristics over the vintages (3 periods).

**Table 1:** demographic and clinical features of study population

Variables	Value n (%)
Mean age	46.47 ± 18.6 years (11-86)
**Gender**	
Male	211 (69.4)
Female	93 (30.6)
**Education level**	
Illiteracy and primary	102 (33.6)
Middle	144 (47.4)
High school and university	58 (19)
**Comorbidities**	
Diabetes mellitus	110 (36.2)
Hypertension	126 (41.4)
Cardiovascular disease	46 (15)
Charlson comorbidity index	3.63±1.8
**Initial nephropathy**	
Diabetic nephropathy	108 (35.5)
Chronic interstitial nephropathy	71 (23.4)
Vascular nephropathy	51 (16.8)
Glomerulonephritis	35 (11.5)
Unknown/other	39 (12.8)
Histories of HD use prior to PD	147 (48.3)
**PD modality**	
Continuous ambulatory peritoneal dialysis	204 (67)
Automated peritoneal dialysis	100 (33)
Nature of PD decision (self/compulsory choice)	262 (86.2) /142 (13.8)
PD assistance	112 (37)

PD: peritoneal dialysis; HD: hemodialysis

**Table 2:** baseline clinical and demographic data of the study patients over the three periods

Variables		Value n (%)		p value
	1990-2000	2001-2006	2007-2017	
Number of patients	47	168	89	
Male gender	40 (85)	116 (69)	55 (62)	0.42
Mean age (years)	44.6 ± 17	48.2 ±18	47.8	0.11
Nature of PD decision (compulsory choice)	8 (17)	3 (2)	4 (4.5)	<0.01
**Education level**				**0.12**
Illiteracy and primary	18 (38.3)	64 (38.1)	20 (22.4)	
Middle	9 (19.2)	28 (16.7)	21 (23.6)	
High school and university	20 (42.5)	76 (45.2)	48 (54)	
PD assistance	20 (42.6)	67 (40)	25 (28)	0.24
Diabetesmellitus	23 (48.9)	58 (34.5)	29 (32.6)	0.13
Hypertension	22 (46.8)	85 (50.6)	19 (21.3)	<0.01
Cardiovascular disease	8 (17)	27 (16.8)	9 (10)	0.32
Previous hemodialysis	17 (36.2)	79 (47)	51 (57.3)	0.05
Predominant primary renal disease	DN: 23 (49)	DN: 57 (34)	DN: 28 (31.5)	0.01
	CIN:11 (23)	VN: 39 (23)	CIN: 25 (28)	
PD Modality CAPD	32 (68)	105 (62.5)	67 (75.3)	0.11
**Time to initiate exchange**				**<0.01**
1 - 14 days	44 (93.6)	122 (72.7)	7 (7.9)	
14 days	3 (6.4)	46 (27.3)	82 (92.1)	
**Type of catheter**				**<0.01**
Flexible Catheter	15 (32)	0	0	
Tenckhoff mono cuff Catheter	32 (68)	168 (100)	10 (11)	
Tenckhoff double cuff Catheter	0	0	79 (89)	
**Pose technique**				**<0.01**
Medical	44 (93.6)	101 (60.1)	0	
Chirurgical mini laparotomy	3 (6.4)	66 (39.3)	89 (100)	
Chirurgical laparotomy	0	1 (0.6)	0	
Occurrence of peritonitis	44 (93.6)	113 (67.3)	49 (55.1)	<0.01
Defaulted aseptic technique	36 (76.6)	96 (57.1)	35 (39.3)	<0.01
**Microorganisms isolated**				**<0.01**
GC+	6 (13.5)	23 (13.6)	21 (24)	
GB-	12 (25.4)	14 (8.5)	13 (14.4)	
Negative culture	23 (49)	128 (76)	52 (58.4)	
Precocious drop out	7 (14.9)	36 (21.4)	30 (33.7)	0.02

PD: peritoneal dialysis; DN: diabetic nephropathy; CIN: chronic interstitial nephropathy; VN: vascular nephropathy; CAPD: continuous ambulatory peritoneal dialysis; GC+: gram-positive cocci; GB-: gram-negative bacilli

**PD related peritonitis: epidemiology and isolated organisms:** seventy eight percent of patients (n=236) presented complications during follow-up and the infectious ones were the most frequent (56%; n=170). Exit site infection and tunnelitis were observed in 32 cases. Four hundred and seventy six episodes of infectious peritonitis were identified among 206 patients. The peritonitis rate (months x patients/peritonitis), as calculated by the peritoneal dialysis and home hemodialysis registry (RDPLF) was 17.6 (0.68 episode per patient-year). Time to occurrence of peritonitis from the start of peritoneal exchange was 14.53 ± 14 months. Most peritonitis cases were observed beyond 3 months (83.9%; n=255). Gram-positive cocci were involved in 54.7% (n=87) of microbiologically confirmed peritonitis cases, mainly caused by *Staphylococcus aureus* (73.6%; n=64). Gram-negative bacilli ranked second (37.7%; n=60), mainly caused by *Pseudomonas* (56.6%; n=34) and *Klebsiella pneumniae* (16.6%; n=10). Peritonitis was fungal in 3.68% of cases (n=1). The rate of culture-negative peritonitis in our series was 65.65% (n=304). The bacterial ecology evolved over the 27 years: predominance of Gram-negative bacilli between 1996 and 2000 (25.4%) then emergence of Gram-positive cocci with constant rise from 2001 till 2017 (13.5 to 24%). The outcome was favorable in 87.6% (n=417) of cases while the PD catheter was removed in the remainder of cases (12.4%; n=59). No significant difference was determined between the peritonitis group and the other group free of peritonitis in term of baseline characteristics neither the PD modality. The prevalence of peritonitis decreased substantially over the years (93.6 to 55.1%) as shown in Table 3.

**Table 3:** comparison of demographic characteristics and clinical data

Variables	Patients with peritonitis	Patients free of peritonitis	p value
Number of patients	206	98	
**Gender**			**0.42**
Male	146 (70.9)	65 (66.3)	
Female	60 (29.1)	33 (33.7)	
Mean age (years)	45.8	47.8	0.11
Residence (urban/rural)	160 (77.7)/46 (22.3)	82 (83.7)/16 (16.3)	0.22
Warm climate	46 (22.3)	28 (28.6)	0.23
**Education level**			**0.86**
Illiteracy and primary	40 (19.4)	18 (18.4)	
Middle	99 (48.1)	45 (45.9)	
High school and university	67 (32.5)	35 (35.7)	
PD assistance	75 (36.4)	37 (37.7)	0.66
Diabetes mellitus	74 (35.9)	35 (35.7)	0.97
Obesity (BMI >25)	2 (1)	0	0.28
**Cause of ESRD**			**0.37**
PD modality CAPD/APD	137 (66.5)/69 (33.5)	67 (68.4)/31 (31.6)	0.74
**Period of PD initiation**			**0.001**
1990 - 2000	43 (20.9)	4 (4)	
2001 - 2006	105 (50.9)	63 (64.3)	
2007 - 2017	48 (23.3)	41 (41.8)	
**Time to initiate exchange**			**0.15**
1 - 14 days	123 (59.7)	50 (51)	
14 days	83 (40.3)	48 (49)	
**Type of catheter**			**0.001**
Flexible catheter	15 (7.3)	0	
Tenckhoff mono cuff catheter	149 (72.3)	61 (62.2)	
Tenckhoff double cuff catheter	42 (20.4)	37 (37.8)	
**Pose technique**			**0.004**
Medical	112 (54.4)	34 (34.7)	
Surgical mini laparotomy	93 (45.1)	64 (65.3)	
Surgical laparotomy	1 (0.5)	0	
Exit site infection/tunnelitis	29 (14.1)	3 (3.1)	0.003

BMI: body mass index; ESRD: end-stage renal disease; PD: peritoneal dialysis; CAPD: continuous ambulatory peritoneal dialysis; APD: automated peritoneal dialysis

**Patient and technique survival:** during the follow-up period, no cases of renal function recovery, indicating PD drop out, were observed. Transfer to HD was the leading cause of dropout (47%; n=143). Thirty patients received renal transplant (RT) and 115 died. The death was unrelated to PD in 74.7% of cases (n=86), mostly due to considerable burden of cardiovascular disease (39.6%; n=34), whereas, sepsis dominates the causes in case of death related to PD (63%; n=17). Ultrafiltration failure was the most important cause of PD withdrawal observed among 43% of the patients (n=60). The statistical study found no significant correlation between ultrafiltration failure and other multiple factors (sex, age, hypertension, heart failure and technical modality). On the other hand, a significant correlation was found with diabetes (p=0.02) and the occurrence of peritonitis (p<0.01). Technique failure causes evolved over the past 27 years, there is an improvement in mortality rate but transfer to hemodialysis is still 10-20% each year; and no statistical difference comparing the 3 periods of the study was found (p=0.6). In contrast, seven covariates presented a significant association with technique failure as shown in the univariate analysis: gender, education level, autonomy, age≥65 years, heart failure, choice and peritonitis. Occurrence of peritonitis was the only independent predictor of technique failure (adjusted relative risk (aRR) 5.07, 95% CI 2.69-9.58) as shown in Table 4.

**Table 4:** univariate and multivariate predictors of technique failure

Variabes	Univariate analysis		Multivariate regression model	
	aRR (95% CI)	P	aRR (95% CI)	P
Gender	1.38 (0.85-2.25)	0.19	0.93 (0.48-1.80)	0.83
Age ≥65 Years	0.41 (0.23-0.76)	0.004	0.76 (0.18-3.23)	0.71
Education level	0.58 (0.36-0.94)	0.03	1.04 (0.049-2.18)	0.91
Heart failure	0.64 (0.34-1.21)	0.17	0.66 (0.23-1.87)	0.44
Choice	2.08 (0.71-6.07)	0.17	2.20 (0.65-7.51)	0.2
PD assistance	1.81 (1.13-2.90)	0.01	2.18 (0.98-4.85)	0.055
Peritonitis	5.62 (3.29-9.58)	<0.001	5.07 (2.69-9.58)	<0.001

PD: peritoneal dialysis; aRR: adjusted risk ratio; CI: confidence interval

The overall non-adjusted patient survival was around 100%, 95% and less than 20% at 1, 4 and 25 years respectively basing on the Kaplan-Meier analysis. The group undergoing renal transplantation had the best survival rate (mean patient survival 291 months ±19) compared to those who received only peritoneal dialysis (aRR 9.926, 95% CI 2.69-9.58; p=0.001). Patients transferred to hemodialysis had an increased survival (aRR 4.498, 95% CI 1.1-18.52; p=0.037) as depicted in Table 5.

**Table 5:** comparison of survival rate and mortality risk between the three groups

	Survival rate (months)			95% CI	
		p	aRR	Inferior limit	Superior limit
Group 1 (PD)	196	0.001	9.926	2.451	40.201
Group 2 (HD)	200	0.037	4.498	1.092	18.52
Group 3 (RT)	291		1		

PD: peritoneal dialysis; HD: hemodialysis; RT: renal transplantation; aRR: adjusted risk ratio; CI: confidence interval

The median technique survival duration was 68 months (95%, CI [47,90]) based on Kaplan-Meier analysis. The survival rates were 75%, 70%, 65%, and 55% at 1, 2, 3 and 5 years respectively. No difference between the two PD modalities was noted (log rank: 0.2). Peritonitis was the primordial predictor of technique survival: For patients presenting peritonitis, the 5-, 10- and 20-year survival rates were 98%, 95% and 65% respectively, compared to 86%, 80% and 20% for the group free of peritonitis (p<0.001).

## Discussion

The PD prevalence in our study was 4.5% of all end stage renal disease patients receiving renal replacement therapy. In Tunisia, although PD gained momentum since it became covered by National Health and Social Security Insurance in 1996, yet the number of patients benefiting from this method remained low: actually, only 224 patients were receiving PD by the end of 2018 according to the summary report by the RDPLF statistics [4]. Our population was relatively younger compared to other populations (mean age of our patients at PD initiation was 46.47 ± 18.6 years) such as the population reported at the 19^th^Annual Report of the Renal Association UK Renal Registry [5,6]. In our series, the two predominant age groups were (20-29) and (50-69) years old. This age bimodal distribution may provide this technique two main strategic orientations: the first, as a bridge towards renal transplantation in young adults. The second, as an at-home autonomous therapeutic method for older patients. Our study revealed an obvious predominance of males, with a sex-ratio (M/F) of 2.27: this difference, concerning end stage renal disease treated by PD, had been reported by multiple other studies [5,6]. The predominant initial nephropathy in our study was diabetic nephropathy (35.5%); while reviewing literature, we found it was variable from a study to another: it ranked second (following chronic interstitial nephropathy), comprising 22.2% in UK renal registry [6], whereas it ranked first in the United States Renal Data System (USRDS) with 37.2% [7].

Peritonitis is still a severe complication and a considerable cause of technique failure and mortality on PD. Prevalence and etiology differ depending on the time period and region. There was an important reduction in the prevalence of peritonitis in our center over this 27-year period. This reduction was associated with better patient compliance for aseptic conditions over time and advances in connecting device technology. However, our peritonitis rate (0.68 episode per patient-year) which was higher than the 0.5 episode per patient-year standard of International Society for Peritoneal Dialysis (ISPD) peritonitis recommendations [8], probably reflecting the low socioeconomic and educational levels of our population. The main cause of peritonitis (54.7%) was gram-positive micro-organisms. Over the years, there was a change in the causative microorganism profile: predominance of gram-negative bacilli between 1996 and 2000, then the emergence of gram-positive cocci with constant progressive rise from 2001 till 2017. Such findings of the predominance of gram-positive bacteria were similar to studies conducted in Scotland, Canada, United States of America and Hong Kong in which gram-positive microorganism comprised for up to 66% of causative pathogens of peritonitis [9-12].

Culture-negative peritonitis was observed in 65.65% of peritonitis cases. It represents a substantial problem which PD centers must face. Recommendations state that culture-negative cases should not soar beyond the threshold of 20% [8,13]. This high frequency of culture-negatives reported in our center incite us towards a better collaboration with our biologist colleagues in order to improve the antibiotic therapy precision and to provide better care for our patients. Traditionally, peritonitis is the main cause of technique failure [14]: this was not different in our study. In fact, it was the reason why 30.1% of patients were definitively transferred to hemodialysis. Peritonitis was the only independent predictor of technique failure in our cohort. However, there were other independent predictive factors found in the literature, such as: age, center experience and automated peritoneal dialysis as initial PD modality [14,15].

In our series, cumulative survival of PD patients was around 100% at 1 year, 95% at 4 years and less than 20% at 300 months (25 years): similar rates were reported in literature [4]. Main causes of mortality were cardiovascular complications (29.6%), then infections (27%), conforming to the results of other studies [14,16]. Concerning the comparison of survival of patients in subgroups hemodialysis and PD, our series demonstrated the superiority of hemodialysis compared to PD. Results are controversial in literature, in terms of survival: Alicia *et al*.based on the UK Renal REIN Registry studied 13767 patients on dialysis, then concluded in support of the planned-hemodialysis [17]. However, in a Chinese study comprising 53,103 patients, Wang I-Kuan *et al*. had reported the interest of using icodextrin to improve survival, especially in diabetic and older patients [18]. The group that initially received PD followed by renal transplantation had the best survival rate (cumulative survival of 90% at 200 months), results conforming to literature [19,20]. Our technique survival rates were lower compared to developed countries [21,22]. Possible explication for our technique survival rates can be associated with health and socioeconomic-related issues: both these problems are affecting survival in developing countries.

Our study proves that the number of patients benefiting from this method in Tunisia remained low and stresses the superiority of hemodialysis compared to PD in terms of survival of patients. The important reduction in the prevalence of peritonitis was associated with better patient compliance for aseptic conditions over time and advances in connecting device technology. On the other side, since this is a retrospective study, we lack adequate data to explore peritoneal membrane transport characteristics and residual renal function mainly described as influencers in several studies. Other prospective studies are needed to establish a support protocol to be based on for better prevention as well as treatment of peritonitis depending on the bacterial ecology of our PD unit.

## Conclusion

Peritoneal dialysis in our center, though the number of patients benefiting from this method was low, is still perceived as an important option for renal failure treatment. We represent a long-term and large description of a successful experience of a single PD center in Tunisia. Peritonitis was independently associated with higher risk of all-cause and infection-related mortality among PD patients and its impact on technique survival was determinate. Despite the gradual decrease of its rate over periods, peritonitis remains frequent in our center and calls for optimization of means of prevention. Ultrafiltration failure seems to be frequent also in our cohort and peritonitis as well as diabetes status were contributing factors. The impactful therapeutic option remains the prevention of these complications. Finally, our study stresses once again the superiority of hemodialysis over PD in terms of overall patient survival.

### What is known about this topic

- Peritoneal dialysis is a useful mode of renal replacement therapy;
- In Tunisia, the number of patients benefiting from this method was low;
- Peritonitis is a severe complication and a considerable cause of technique failure and mortality on peritoneal dialysis; the comparison of survival of patients in subgroups HD and PD was controversial.

### What this study adds

- The number of patients benefiting from this method in Tunisia remained low;
- The important reduction in the prevalence of peritonitis was associated with better patient compliance for aseptic conditions over time and advances in connecting device technology;
- Superiority of HD compared to PD in terms of survival of patients.
